# Effectiveness of a community health worker-led case management programme to improve outcomes for people with psychotic disorders in Thailand: a one-year prospective cohort study

**DOI:** 10.1186/s12888-022-03888-1

**Published:** 2022-04-08

**Authors:** Tawanchai Jirapramukpitak, Kankamol Jaisin, Suttha Supanya, Patcharapim Takizawa

**Affiliations:** 1 Institute for Population and Social Research, Mahidol University, Nakhon Pathom, Thailand; 2 Centre of Excellence in Applied Epidemiology, Thammasat University, Pathumthai, Thailand; 3 Centre for Global Mental Health, Institute of Psychiatry, Psychology and Neuroscience, King’s College London, London, UK; 4 Department of Psychiatry, Faculty of Medicine Siriraj Hospital, Mahidol University, Bangkok, Thailand; 5 Department of Mental Health, Ministry of Public Health, Somdet Chaopraya Institute of Psychiatry, Bangkok, Thailand; 6 Department of Global Public Health, Faculty of Medicine, University of Tsukuba, Tsukuba, Ibaraki Japan

**Keywords:** Community health services, Early intervention, Low and middle-income countries, Observational studies, Mental health, Propensity score

## Abstract

**Background:**

Intensive case management (ICM) programmes for psychotic patients are effective in improving outcomes, but often unfeasible in resource-poor settings, as they typically require extensive human resources and expertise. We developed and evaluated the effectiveness of a less intensive case management program (LICM), led by community health workers, on one-year social functioning and service use.

**Methods:**

A prospective cohort study was conducted on patients aged 18 and above residing in a hospital catchment area. Outcomes were compared between LICM (*n* = 64) and non-LICM participants (*n* = 485). A counterfactual framework approach was applied to assess causal effects of the LICM on outcomes. The programme effectiveness was analyzed by augmented-inverse probability of treatment weighting (AIPW) to estimate potential outcome mean (POM) and average treatment effect (ATE). Outcomes were employment status and use of emergency, inpatient and outpatient services. Analyses were stratified by the number of previous psychotic relapse (≤ 1, > 1) to assess heterogeneity of treatment effect on those in an early and later stages of psychotic illness.

**Results:**

In the early-stage cohort, the likelihood of being employed at one year post-baseline was significantly greater in LICM participants than non-LICM participants (ATE 0.10, 95%CI 0.05–0.14, *p* < 0.001), whereas service use of all types, except outpatient, was not significantly different between the two groups. In the later-stage cohort, the likelihoods of employment between the two groups at post-baseline were similar (ATE -0.02, 95%CI -0.19–0.15, *p* = 0.826), whereas service use of all types was significantly higher in LICM participants.

**Conclusion:**

LICM in a setting where community mental services are scarce may benefit those at an early stage of psychotic illness, by leading to better social functioning and no higher use of unscheduled services at the end of the programme, possibly through their better prognosis and medication adherence. A more intensive case management model may be appropriate for those in a later stage of the illness.

**Supplementary Information:**

The online version contains supplementary material available at 10.1186/s12888-022-03888-1.

## Introduction

Psychotic disorders contribute to a substantial burden on society. In Thailand, the economic cost of schizophrenia, a common type of psychotic disorder, is estimated to be around 1,000 million USD) annually [1]. To put this into context, the cost of this illness alone is about 15% of Thailand’s total annual universal health care budget of 6,600 million USD in 2022 Although considerable progress has been made in terms of service availability, high unmet needs and low treatment rates persist among adults with psychosis [2]. The problem is also compounded by treatment drop-out and poor medication adherence, leading to poor outcomes in the longer term.

In Thailand, low rates of service use among psychotic patients are a major concern. In some catchment areas, as low as 11.5% of psychotic patients sought treatment [3]. Treatment dropout is also common. Studies show that dropout from outpatient mental healthcare in the past 12 months range from 37–45% in low- and middle-income countries [4]. Thus, a large share of patients will receive no or inadequate treatment if these challenges remain unmet. To address such problems, a model of care called case management (CM) has been advocated as an effective treatment program [5, 6]. CM is a community-based package of care, delivered by a team of health professionals and designed to meet the needs of these patients [7]. Evidence to date suggests that Intensive Case Management (ICM), a variant of CM, which emphasizes the importance of small caseload, individualized services, frequent contact and high-intensity input, is effective in ameliorating many outcomes such as reducing hospitalization, increasing retention in care and improving social functioning, compared to standard community care [8], especially where standard community care is under-resourced [9]. However, evidence suggests that uncertainty remains over whether ICM is more effective than less intensive CM. It is recommended that more studies on the efficacy of less intensive CM compared with standard care be undertaken [8].

ICM also has some potential drawbacks. It is usually designed for a small proportion of patients with severe mental illness, which can potentially leave a large proportion of less severe mentally ill patients in inadequate care and higher risk of relapses, and consequentially put them at risk of more serious or chronic illness in the longer term [10]. ICM also relies on great use of personnel and resources, making it difficult for the vast majority of affected individuals in low-resource settings to receive such model of care.

In ICM models, health professionals with professional qualifications typically act as case managers [11]. Nevertheless, ‘community carers’ with relatively little formal training are often used to provide direct care to the long-term mentally ill [12]. There is also growing evidence that community health workers can improve access to and use of mental health and social services [13]. Recent work in India adapted the CM model to be led by community health workers (CHWs), who work collaboratively with specialists in facility-based settings, and found that the model was modestly more effective than the facility-based model alone in reducing symptoms of psychosis [14]. As in Thailand, simple home-based interventions are often delivered by CHWs, locally known as village health volunteers (VHVs). They constitute a large part of the country’s primary healthcare system, playing a crucial role in basic and continuing care and making health services more available, accessible and acceptable. There are approximately a million VHVs across the country, each working with seven to twelve families in most communities [15]. Integrating CHWs into case management work, therefore, offers the opportunity not just to make this approach more feasible and appropriate in resource-limited settings but to truly impact the outcomes of some of the most difficult and hard to reach individuals with mental illness.

### Development of a less-intensive CM model, led by community health workers, for people with psychotic disorders

The Thai mental health care system is, to a large extent, still hospital-based. The first publicly-funded case management program for mentally ill patients in Thailand, called continuity of care (CoC), was recently launched in 2016 by the National Health Security Office (NHSO) in some selected areas [16]. The scheme was originally intended for patients with schizophrenia and other psychotic disorders more generally, but has now evolved to more specifically target those with serious mental illness (SMI) or living in challenging circumstances.

There are two main groups of local staff running the CoC programme, namely mentoring and caring teams. The mentoring team consists of specialists working in major provincial or psychiatric hospitals i.e. consultant psychiatrists or senior nurses. The caring team consists of nurses in subdistrict hospitals or primary care units, working in collaboration with CHWs in the communities. The programme’s primary goals were to improve adherence to medication and regular follow-ups. Home visits are usually made once a month or less.

The scheme, however, has limitations. It is designed to keep patients who are in current contact with mental health services in continuing care, effectively excluding those who have never sought treatment, lost contact with mental health services, are geographically mobile, or are seeking treatment outside the national universal health care scheme. The CoC programme has yet to be properly evaluated for its effectiveness.

To address those limitations and gaps in knowledge on the effectiveness of the CoC model, we developed a collaborative case management program led by CHWs, but less intensive than the usual ICM model (hereafter called LICM) for people with psychotic disorders. The LICM is built upon the CoC, but primarily differs from the CoC in that it adopts a more assertive and inclusive approach, such as active case identification in the community, more engagement with patients and families, and encompassing of patients under any (or no) health coverage scheme. LICM requires less resource input than ICM as community health workers themselves, rather than qualified mental health professionals, act as a case manager, and work in collaboration with hospital-based specialists in their catchment area. It provides services to patients with mental illness of varying degree of severity, not only SMI. It does not offer treatments in the community, which usually require highly-skilled professionals to deliver (such as rehabilitation, psychotherapy or social skill training).

We used a prospective cohort study design with a counterfactual framework approach to determine the treatment effect of LICM by comparing treatment outcomes between the LICM-attending sample and a matched non-LICM sample at the end of the one-year study period. The counterfactual approach evaluates treatment effectiveness in a situation where an RCT cannot be conducted for ethical or cost reasons [17]. We applied this method to answer our research questions—whether the LICM programme would improve social functioning and service use outcomes in patients in different stages of psychotic illness.

## Materials and methods

This was a 12-month naturalistic, prospective observational cohort study. The study was approved by the Ethics Committee of Thammasat University (No 096/2560).

### Setting

The study was conducted on a sample of the non-institutionalized adult population aged 18 or older residing in a catchment area served by a university hospital. It was a typical mixed used commercial and residential urban area in the north of the Bangkok Metropolitan Region. The study setting was not currently served by the CoC or any other case management programmes.

### Recruitment of study participants

As a substantial number of psychotic patients have no previous or current contact with psychiatric services, it is therefore essential to identify and include such groups of patients in the study to maximize the inclusiveness and representativeness of the psychotic patient population. Not only did we identify potentially eligible patients through the medical records of those who had been treated for psychotic disorders (ICD-10, F20-29) at the coordinating university hospital and its affiliated primary care unit, we also did some form of community outreach to identify those who had no current contact with the services, but may need psychiatric care. These patients were recruited from the following sources: 1) those who had reported a lifetime history of psychotic symptoms in a survey conducted in the same catchment area in 2015–2016 [10], and 2) those who had been identified by key informants in the community using a psychosis screening questionnaire (Mongkol et al. 2000) through a process modified from the chain referral sampling method [18, 19].

### Description of the service programme

Given the resource constraints and the practicality of delivering the LICM programme by available community health workers, not all patients were offered the service. Selection criteria tended to be based on patients’ symptoms and treatment compliance. Specifically, those with current severe symptoms, poor adherence to medication, problem behaviors (such as substance use or harmful behavior), and no or little support from family members were more likely to be selected for the programme. Differences between LICM and non-LICM services were summarized in Table 1.

**Table 1:** LICM compared to non-LICM service

	**LICM**	**Non-LICM**
**Health service provision**	Inpatient, outpatient, emergency	Inpatient, outpatient, emergency
**Workers involved in care**	1.Hospital staff (consultant psychiatrist, psychologist, psychiatric nurse, social worker) 2.Programme coordinators 3.Community health workers	Hospital staff (consultant psychiatrist, psychologist, psychiatric nurse, social worker)
**Main health care workers**	Community health workers in collaboration with hospital staff	Only hospital staff
**Work style**	Individual caseload	Individual caseload
**Engagement**	Assertive; focus on engagement and medication adherence, multiple attempts	Non-assertive, no follow-up of missed appointments/reports of non-compliance
**Site of most visits**	Home visits	Office-based
**Main intervention**	Home visit	No home visit
**Assistance with engagement in treatment**	All received the assistance if necessary (e.g. medical appointment, accompanied transport to hospital, ambulance service, bringing medicine from hospital to patients)	Few received such assistance
**Assistance with access to social service**	All received the assistance if necessary e.g. disability benefit, disability health coverage)	Few received such assistance
**Working hours**	Flexible, often after-hour service	Office hours (after-hour service provided by hospital emergency unit only
**Frequency of contacts**	Individualized according to patient need; weekly to monthly	Monthly to three-monthly

#### LICM

The programme was a collaborative effort and provided by three sets of persons. First, the hospital staff team, consisting of a psychiatrist, a clinical psychologist, and a nurse, provided clinical leadership and supervision, advice on necessary pharmacological treatment and treatment planning. Second, two program coordinators, who were lay health workers with experience of working with CHWs in the catchment communities, supervised the CHWs, coordinated the programme, and made assessments at baseline and one-year endpoint. Weekly meetings between program coordinators and hospital staff were held to discuss issues throughout the study period. Third, eleven CHWs, acting as a case manager, each worked with 2–10 patients and their caregivers at home. They were responsible for making home visits, providing psychoeducation, managing medication adherence, arranging medical appointments, accompanying patients to hospital (if necessary), and, if requested, facilitating access to disability benefits and disability health coverage. The home visits were conducted weekly, forthnightly or monthly as appropriate during the one-year service period. A home-visit form was used to record changes in nine important domains on each visit, including the presence and frequency of psychotic symptoms, level of medication adherence, availability of supportive caregiver, level of self care, job capacity, quality of relationship with family members, living condition, communication and learning ability [20]. Efforts were made to maximize family involvement by scheduling visits at their convenience and informing them well in advance of proposed visits, and to minimise the possibility of unwitting illness disclosure by emphasizing the ethics and importance of keeping patients’ confidentiality by the CHWs. Monthly meetings between case managers, program coordinators and hospital staff were held monthly for 60–90 min at the hospital coordinating centre. The issues discussed included care plan, treatment adherence strategies, and problems in managing difficult patients.

#### Non-LICM (control)

The control cohort did not receive the LICM package as described above, although small proportions of the control group received some parts of the programme. For example, a few received assistance in facilitating accompanied transport to hospital (5.36% of the non-LICM vs. 70.31% of the LICM-cohort), in access to disability benefits (1.86% vs. 34.38%), and in bringing medicine from hospitals (0.41% vs. 21.88%). No one in the control group received the home visit component.

### Measurement

Baseline data on socioeconomic characteristics, psychotic symptoms, illicit drug use and service utilization were collected from July 2017 until September 2017, using part of the World Mental Health Survey version of the World Health Organization-Composite International Diagnostic Interview (WMH-CIDI), a fully-structured lay administered diagnostic interview, to assess history of psychotic symptoms and service utilization [21]. The instrument was also used in a recent national mental health survey in Thailand [2]. The first participant started attending the program in October 2017 and the last in June 2018. The programme ended in May 2019, lasting about a year for each participant. Participants in the control group were contacted about 12 months post-baseline for endpoint assessment.

### Control variables

There were 2 sets of covariates, one for treatment model and the other for outcome models. Baseline covariates for the treatment model were selected to compare the LICM sample with the crude non-LICM sample, based on theoretical associations with both the assignment to the treatment and the outcome variables, following the recommendations of Garrido and colleagues [22]. These included age (continuous), baseline paid employment, current presence of active psychotic symptoms, one-year history of illicit drug use and lifetime history of hospital admissions due to psychiatric illness. Employment was used as a proxy for social functioning and symptom levels, because working is highly correlated with such outcomes [23]. Covariates for the outcome models were based on the treatment model above but also included age at onset of psychosis and the number of lifetime psychiatric hospitalizations. Age at onset is a well-known predictor of long-term prognosis [24], whereas the number of past admissions also influences current social functioning and service utilization [25].

### Outcome variables

Two major outcome domains, namely employment status and service use over the one-year post-baseline period, were used to compare the LICM and non-LICM cohorts. The two domains are commonly used to establish the effectiveness of care [26].

Employment was defined as respondents’ reporting of 12-month personal earnings before taxes (counting only wages and other stipends from employment and excluding pensions, investments, or other financial assistance). The presence of such earnings was classified as being employed.

Service use included number of outpatient visits that involved psychotropic drug prescriptions, number of psychiatric admissions and their total length (in days), and number of emergency room (ER) visits for psychiatric reasons throughout the one-year study period. These types of service use are often used to reflect clinical outcomes and social costs [26]. In this study, hospitalization decisions were made jointly by the patients’ family members and hospital staff based primarily on the severity of their symptoms: the project teams did not unilaterally admit or send patients to hospital.

### Statistical analysis

Characteristics of study participants were described using mean (SD) and frequency (percentage) for continuous and categorical data, respectively. To estimate treatment effects, we used propensity scores to balance the distribution of patient characteristics in the two treatment groups to reduce bias due to non-random assignment to treatment group. The scores were estimated and the balancing property was checked. The treatment model was constructed by fitting treatment assignment variable (i.e., LICM vs non-LICM) on the selected baseline covariates using logit equation. The outcome models were constructed using either a logit or poisson equation, depending on whether the outcome was binary or count data, by fitting each of the outcome variables on the covariates. We then performed a doubly robust estimation by employing the augmented-inverse probability of treatment weighting (AIPW) techniques. AIPW was applied to estimate potential outcome mean (POM), that is, the predicted mean associated with the LICM cohort relative to the non-LICM cohort, taking into account the propensity to be in the LICM group. Based on the POM, average treatment effect (ATE) was calculated for each outcome, which was the difference in the post-baseline outcome unit of measurement for those who participated in the LICM compared to what they would have experienced if they had not participated in the LICM. ATEs were calculated for the entire sample, and separately for early (having ≤ 1 previous psychotic relapse) and later (having > 1 previous psychotic relapses) stages of illness to explore potential differences of illness stage in the impact of LICM, and the errors associated with those estimated ATEs. We considered this early-stage population to be important due to their potentially better treatment outcomes [27, 28]. We used Stata version 14.2 [29] with teffects aipw for estimating treatment effects. Statistical significance was set at the 5% level.

## Results

### Sample description

**Fig. 1:**
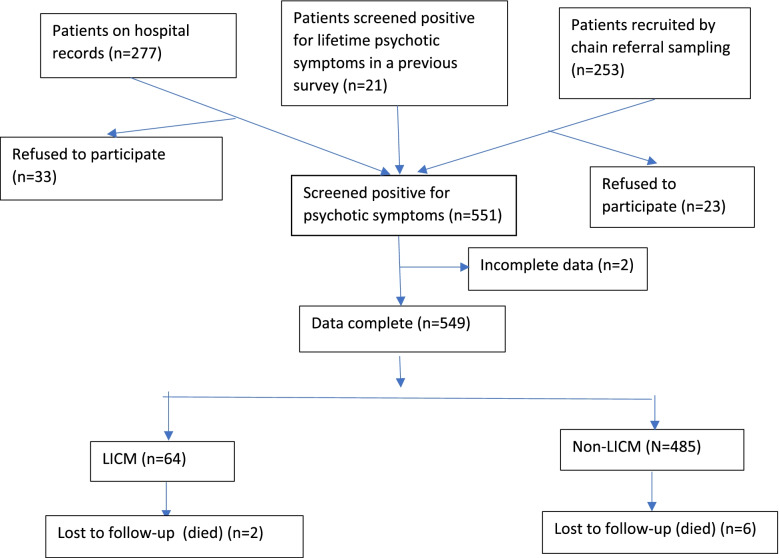
Flow diagram of study enrollment, allocation and follow-up

Flow diagram of study enrollment, allocation and follow-up was shown in Fig. 1. The majority of the sample were identified and recruited by contacting those who had been residing in the hospital catchment area and seeking treatment at the university hospital and its affiliated primary care unit (PCU) (*n* = 277). The second majority were identified and recruited by known informants using the chain referral sampling method (*n* = 253). The remaining were those who had been screened positive for psychotic symptoms in the earlier conducted catchment area survey and had no history of receiving treatment at the coordinating hospital and its PCU (*n* = 21). The three sources constituted a total of 551 participants. Two participants had incomplete data and were thus excluded, leaving a total of 549 participants for inclusion in the analysis: 64 patients in the LICM cohort and 485 patients in the non-LICM cohort. About 1.5% (*n* = 8) had died by the end of the follow-up period: 3.1% (*n* = 2) of the LICM arm and 1.2% (*n* = 6) of the non-LICM arm.

Table 2 shows the baseline descriptive characteristics of LICM and non-LICM recipients. The sample was predominantly female (51.0%) with a mean age of 49.8 years. The majority were single (40.0%) and completed primary school education or less (88.2%). Less than half had been employed in the previous year (45.9%). Around one-fourth (26.2%) reported current presence of psychotic symptoms, and 29.1% had a history of psychiatric hospitalization. Around 32% had suffered more than 1 psychotic relapse and were thus classified as the later-stage group.

**Table 2:** Descriptive characteristics of the baseline LICM and non-LICM cohorts

**Characteristics**	**Total** **(*N* = 549)**		**Non-LICM (*N* = 485)**		**LICM (*N* = 64)**		***P***
	**N**	**%**	**N**	**%**	**N**	**%**	
**Gender**							
Male	269	49.0	235	48.5	34	53.1	
Female	280	51.0	250	51.6	30	46.9	0.482
**Age (mean, sd)**	49.8 (15.1)		50.7 (15.0)		42.6 (13.3)		< 0.001
**Education**							
≤ primary school	484	88.2	433	89.3	51	79.7	0.026
> primary school	65	11.8	52	10.7	13	20.3	
**Marital status**							
Married	180	32.8	168	34.6	12	18.8	< 0.001
Separated/divorced/widowed	155	28.2	144	29.7	11	17.2	
Single	214	40.0	173	35.7	41	64.1	
**Baseline employment**							
Employed	252	45.9	237	48.9	15	23.4	< 0.001
Unemployed	297	54.1	248	51.1	49	76.6	
**Current psychotic symptoms**							
Absent	405	73.8	381	78.6	24	37.5	< 0.001
Present	144	26.2	104	21.4	40	62.5	
**History of psychiatric hospitalization**							
No	400	72.9	375	77.3	25	39.1	< 0.001
Yes	149	29.1	110	22.7	39	60.9	
**Psychiatric hospitalization in the past year**							
No	487	96.1	435	98.0	52	82.5	< 0.001
Yes	20	3.9	9	2.0	11	17.5	
**Illicit drug use**							
No	501	91.3	452	93.2	49	76.6	< 0.001
Yes	48	8.7	33	6.8	15	23.4	
**Number of past psychotic relapse**							
≤ 1 (early stage)	372	67.8	346	71.3	26	40.6	< 0.001
> 1 (later stage)	177	32.2	139	28.7	38	59.4	

Compared with non-LICM participants, LICM patients were younger (42.6 vs. 50.7 years), predominantly male (53.1% vs. 48.5%), better educated (10.1 vs. 8.2 years of schooling), predominantly single (64.1% vs. 35.7%), more likely to report current presence of active psychotic symptoms (62.5% vs. 21.4%) and to have used illicit drugs in the previous year (23.4% vs. 6.8%). The LICM cohort was also more likely to be unemployed in the past year (76.6% vs. 51.1%), to have a lifetime (60.9% vs. 22.7%) and one-year (17.5% vs. 2.0%) histories of psychiatric hospitalization, and to have experienced 2 or more previous psychotic relapses (59.4% vs. 28.7%).

### Main analysis

The multivariate logit model showed that baseline employment, current presence of psychotic symptoms, and history of psychiatric hospitalization were statistically associated with LICM (Table S 1). These variables were therefore considered in the treatment model. Age and illicit drug use were not significantly associated with LICM assignment but were nevertheless included in the model because they were theoretically thought to affect the assignment outcome.

The overlap assumption for applying the treatment effect model was checked by plotting estimated densities of predicted probability against propensity score (Figure S 1). The plot indicated that the predicted probability that LICM attendants were assigned to LICM and the predicted probability that non-LICM attendants were assigned to LICM considerably overlapped. Thus, the overlap assumption was largely observed.

### Average treatment effects of the LICM programme on outcomes

Significant ATEs of LICM were found on service use (Table 3) when all stages of illness were combined, indicating that on average and after adjusting for baseline covariates, participants who took part in the LICM programme more significantly utilized inpatient (ATE 0.26, 95%CI 0.03–0.49, *p* = 0.028), outpatient (ATE 2.38, 95%CI 1.45–3.30, *p* < 0.001) and emergency (ATE 0.15, 95%CI 0.04–0.26, *p* = 0.010) services than those who did not participate in the programme.

**Table 3:** ATEs of receiving LICM on outcomes one year post-baseline using AIPW, stratified by stage of illness^a^

Main outcome^b^	All stages (*n* = 549)					Early stage (*n* = 372)					Later stage (*n* = 177)				
	ATE			POM		ATE			POM		ATE			POM	
	Coefficient	95%CI	*P* value	Non-LICM	LICM	Coefficient	95%CI	*P* value	Non-LICM	LICM	Coefficient	95%CI	*P* value	Non-LICM	LICM
**Employment**	0.03	-0.11–0.17	0.686	0.34	0.37	0.10	0.05–0.14	< 0.001	0.40	0.49	-0.02	-0.19–0.15	0.826	0.23	0.21
**Number of psychiatric admission**	0.26	0.03–0.49	0.028	0.03	0.29	0.10	-0.01–0.22	0.064	0.04	0.14	0.41	0.08–0.75	0.016	0.01	0.43
**Total length of hospital stay (days) due to psychiatric reasons**	4.97	0.56–9.39	0.027	0.79	5.77	2.55	-5.56–10.66	0.538	0.98	3.53	9.37	1.68–17.05	0.017	0.28	9.65
**Any psychiatric ER visit**	0.15	0.04–0.26	0.010	0.02	0.17	0.12	-0.02–0.25	0.100	0.02	0.14	0.19	0.03–0.36	0.023	0.03	0.23
**Number of outpatient visit**	2.38	1.45–3.30	< 0.001	2.97	5.34	2.55	0.08–5.03	0.043	3.04	5.60	3.23	2.03–4.43	< 0.001	2.77	6.00

*POM* Potential Outcome Mean, *ATE* Average Treatment Effect

^a^ Stage of illness is classified as early (having ≤ 1 past psychotic relapse) and later stages (having > 1 past psychotic relapses)

^b^ Values were adjusted for baseline employment, age, current presence of active psychosis, illicit drug use, age at onset of psychosis, number of past psychiatric admission

After stratifying by stage of illness, significant ATE on employment was found in the early-, but not in the later-stage psychosis. The estimated ATEs (i.e., risk differences) of LICM on being employed at one-year post-baseline were 0.10 (95%CI 0.05–0.14, *p* < 0.001) and -0.02 (95%CI -0.19–0.15, *p* = 0.826) among those in the early- and later-stage psychosis, respectively. The POMs of being employed post-baseline were 0.40 (95%CI 0.34–0.45) and 0.49 (95%CI 0.44–0.55) in the non-LICM and LICM early-stage cohorts, respectively. The change in ATE between the LICM and non-LICM participants’ POMs when this early stage of illness is concerned indicated that for every 10 early-stage psychotic individuals attending the one-year LICM programme, one person could avoid transitioning into unemployment (i.e. number needed to treat).

On the other hand, the ATE estimates of LICM on all service use outcomes were substantially higher among the later-stage cohort than the early-stage cohort. In the later-stage cohort, the ATE of LICM was 0.41 (95%CI 0.08–0.75, *p* = 0.016) on the number of psychiatric inpatient admission, 9.37 (95%CI 1.68–17.05, *p* = 0.017) on the total length of hospital stay, 0.19 (95%CI -0.03–0.36, *p* = 0.017) on any psychiatrically-related ER visit, and 3.23 (95%CI 2.03–4.43, *p* < 0.001) on the number of outpatient visit. All these estimates indicated significant changes in POM i.e. the mean number of visits for each type of service use or the number of days admitted in hospital for length of stay. In the early stage cohort, of all types of service use, only outpatient use was significantly higher in the LICM arm than the non-LICM arm (ATE 2.55, 95%CI 0.08–5.03, *p* = 0.043).

## Discussion

The study supports the feasibility of our less intensive case management model led by community health workers, and adds to a growing evidence of the significant roles of CHWs in delivering mental health interventions and in improving mental health equity among people with mental illness, especially those from underserved populations [30].

Essentially, we found that the impacts of attending LICM on outcomes were different for early- and later-stage cohorts. The finding that LICM was effective in maintaining social functioning by keeping more patients in employment over time, but only among those in the early stage of psychotic illness, corresponds with the notion that early psychosis intervention programmes may provide better longer term treatment outcomes than usual care. A meta-analysis of randomized clinical trials found that early intervention services, which usually included CM components, led to better clinical outcomes, including involvement in school or work, for patients with early phase psychosis compared with treatment as usual, (relative risk = 1.13; 95%CI 1.03–1.24) [28]. LICM was potentially beneficial for this group partly because it facilitated access to psychiatric services, as shown, for example, by an increased use of outpatient service after attending the programme (i.e. more regular follow-up), and leading possibly to improved medication adherence.

A plausible explanation why there was no apparent benefit of LICM in increasing the likelihood of employment among the later-stage cohort was that their course of illness had already passed a critical stage, thus making it more challenging for them to recover. Longitudinal studies of patients with psychosis reported that the duration of active psychosis after treatment (DAT), which included that of relapses, was one of the best predictors of long-term social functioning [31, 32]. This means that for multiple-episode or chronically psychotic patients, more intensive CM may be more appropriate. Evidence points in the direction that that more intensive CM, especially one that is more adherent to the assertive community treatment (ACT) model, is better at improving outcomes in those with serious mental illness [8].

In terms of service use, LICM did not generally significantly reduce use of unscheduled services. Instead it even significantly increased use of such services in the later-stage cohort. The increased use, for example, of inpatient services among CM-attendants was also sometimes observed elsewhere [33]. This could be explained by a few reasons. First, evidence suggests that case management could decrease the use of inpatient services, particularly if the use it is high before the programme enrollment (i.e. at least 6 days of hospital stay per month in the previous two years) [8], while in the present study, the average inpatient service use at baseline among LICM participants was relatively low (i.e. only 0.35 and 7.81 days of hospital stay for the whole year in the previous year in the early- and later-stage LICM cohorts, respectively) (Table S 2). Second, it is unlikely that case management will reduce rehospitalization rates unless appropriate and effective outpatient and community services are available, because rehospitalization is often the only treatment alternative if other options are not available [33], which was more often than not found to be the case in our study setting.

LICM also generated significantly more outpatient use in both early and later-stage cohorts than non-LICM. At post-baseline, the potential outcome means of outpatient use in the LICM groups were considerably higher (i.e. 5.6 and 6.0 visits a year in the early- and later-stage cohorts) than the non-LICM groups (i.e. 3.0 and 2.8 visits in the early- and later-stage cohorts). It is generally recommended that a minimum of 4 visits per year is required for follow-up and medication monitoring during a course of treatment for psychotic disorders in evidence-based treatment guidelines [34, 35]. LICM successfully followed the recommendation by achieving the recommended number of outpatient visits. It appeared that without LICM, patients would be less likely to receive adequate care and treatment.

From public health and economic perspectives, for every 10 early-stage psychotic individuals attending the one-year LICM programme, one person could avoid becoming unemployed. On a longer term, this could potentially reduce economic burden due to otherwise growing unemployment over time among a large proportion of people with chronic mental illness [10]. Moreover, the programme did not incur additional costs by significantly increasing the use of inpatient and emergency services. These potential benefits seem to be in consistent with many economic evaluations of early intervention services, which reported that the services are cost-effective, and even more so when considering societal costs [36].

The strengths of this study include the use of a prospective cohort community sample with low levels of attrition. The longitudinal design enabled the adjustment of the baseline characteristics for each individual participant. The sample was recruited from a variety of sources, not just those currently in contact with services, to increase the inclusiveness and representativeness of the psychotic populations. The main data collection tool, WMH-CIDI, is also a widely-used and valid instrument for assessing mental health problems and care utilization in a large epidemiological survey. The quality of data in this study was therefore likely to be better than routinely obtained medical data used in many studies.

The study also has a number of potential limitations. The findings were based on data from a relatively small number of program patients. Some outcomes had few events observed, thereby limiting the robustness of some results. The generalizability is potentially limited by setting and operator variables. Detailed information on psychotic symptoms and medication adherence were not available, precluding assessment of LICM impact on mental health symptom outcomes, although baseline and post-baseline employment should act as a reasonable proxy for symptom severity. In addition, despite controlling for a number of important differences between the program and nonprogram cohorts, these results remain subject to residual confounding. A randomized control trial design would be the standard approach to address this issue.

### Implications for mental health services

The findings provide potential policy implications in terms of designing of case management programs that are potentially cost effective, feasible and appropriate to the context and to each stage of psychotic illness. First, our LICM model does not use extensive resources and may be a better use of resources than the ongoing CoC model currently implemented in the country. Doubts remain over the effectiveness of the CoC model because, generally, it is even less intensive than our LICM model but tends to targets patients with a higher severity or complexity level.

Second, the findings confirm the crucial role of case management in early intervention services, because it allows greater opportunities to engage patients early in the course of their illness and to continually monitor these patients. These are among the key factors that drive more favourable outcomes [37].

Third, compared with other CM models, LICM is more flexible because CHWs in the Thai and perhaps many other contexts provide care for community-dwelling people in general, whether or not they are physically or mentally ill. It therefore has an added advantage in that, in many cases, it could potentially reduce the stigma associated with mental health care facilities.

As with all observational studies, the findings of the present study may provide important questions to be tested in randomized trials and cost-effectiveness studies. Future studies with larger samples that follow-up patients for a longer period are crucial. Data on the contributions of the LICM programme to quality of life, family burden and other outcomes such as criminal involvement and physical harm to self and others, would be especially valuable.

## Conclusions

Our less-intensive, CHW-delivered CM model have a potential role in improving psychotic case identification, access to services, engagement in services and reducing a decline in social functioning without resulting in higher use of unscheduled services in early-stage psychotic patients. It is potentially more cost effective in settings with limited resources. More intensity CM may be more appropriate for patients with a later stage of psychotic illness. The ongoing CM-based CoC programme for people with serious mental illness currently in place in Thailand may not be highly beneficial in improving social functioning and service use outcomes among this group.

## Supplementary Information

**Additional file 1:Table S1.** Factors associated with LICM assignment: a multivariate logistic model. **Figure S1. **Distribution of propensity scores. **Table S2.** Information on service use at baseline by stage of illness. **Table S3.** Information on employment and service use at one-year pos-baseline period.

## Data Availability

The dataset used and/or analysed during the current study are available from the corresponding author on reasonable request.
