# Supplementary material for: Effectiveness of a community health worker-led case management programme to improve outcomes for people with psychotic disorders in Thailand: a one-year prospective cohort study
Source: BMC Psychiatry. 2022 Apr 8;22:247. doi: 10.1186/s12888-022-03888-1 (PMC8991661; doi:10.1186/s12888-022-03888-1)
Supplement: Supplementary file 1 — Additional file 1:Table S1. Factors associated with LICM assignment: a multivariate logistic model. Figure S1. Distribution of propensity scores. Table S2. Information on service use at baseline by stage of illness. Table S3. Information on employment and service use at one-year pos-baseline period. [file 12888_2022_3888_MOESM1_ESM.docx]

# Supplementary material

**Table S1** Factors associated with LICM assignment: a multivariate logistic model

| Factors | Coefficient | Standard error | p-value | Odds ratio (95%CI) |
| --- | --- | --- | --- | --- |
| **Age** | -0.02 | 0.01 | 0.105 | 0.98(0.96-1.00) |
| **Employment status** | -0.74 | 0.35 | 0.034 | 0.48 (0.24-0.95) |
| **Presence of active psychotic symptoms** | 1.76 | 0.31 | <0.001 | 5.84(3.15-10.80) |
| **Use of illicit drug** | 0.62 | 0.41 | 0.136 | 1.84 (0.83-4.12) |
| **History of psychiatric admission** | 1.47 | 0.33 | <0.001 | 4.34(2.27-8.29) |

**Figure S1** Distribution of propensity scores

**Table S2** Information on employment status and service use at baseline by stage of illness

| Characteristics | Early stage (n=372) | | | | | | | Later stage (n=177) | | | | | | | |
| --- | --- | --- | --- | --- | --- | --- | --- | --- | --- | --- | --- | --- | --- | --- | --- |
|  | Non-LICM | | LICM | | Total | | | | Non-LICM | | | LICM | | Total | |
|  | N | % | N | % | N | | % | | N | % | | N | % | N | % |
| **Employment** |  |  |  |  |  | |  | |  | |  |  |  |  |  |
| No | 160 | 46.2 | 19 | 73.1 | 179 | | 48.1** | | 88 | | 63.3 | 30 | 79.0 | 118 | 66.7 |
| Yes | 186 | 53.8 | 7 | 26.9 | 193 | | 51.9 | | 51 | | 36.7 | 8 | 21.1 | 59 | 33.3 |
| **History of psychiatric hospitalization** |  |  |  |  |  | |  | |  | |  |  |  |  |  |
| No | 280 | 80.9 | 10 | 38.5 | 290 | | 78.0* | | 95 | | 68.4 | 15 | 39.5 | 110 | 62.2* |
| Yes | 66 | 19.1 | 16 | 61.5 | 82 | | 22.0 | | 44 | | 31.7 | 23 | 60.5 | 67 | 37.9 |
| **Psychiatric hospitalization in the past year** |  |  |  |  |  | |  | |  | |  |  |  |  |  |
| No | 342 | 98.8 | 23 | 88.5 | 365 | | 98.1* | | 134 | | 96.4 | 30 | 79.0 | 164 | 92.7* |
| Yes | 4 | 1.2 | 3 | 11.5 | 7 | | 1.9 | | 5 | | 3.6 | 8 | 21.1 | 13 | 7.3 |
|  |  |  |  |  |  | |  | |  | |  |  |  |  |  |
|  | Mean | SD | Mean | SD | Mean | | SD | | Mean | | SD | Mean | SD | Mean | SD |
| **Number of lifetime psychiatric admission** | 0.29 | 0.69 | 0.85 | 0.78 | 0.33 | | 0.71* | | 0.48 | | 0.80 | 1.13 | 1.04 | 0.62 | 0.90* |
| **Total length of stay for psychiatric reasons in the lifetime** | 1.85 | 7.48 | 2.81 | 4.92 | 1.92 | | 7.33 | | 2.45 | | 10.94 | 13.47 | 24.57 | 4.81 | 15.54* |
| **Total length of stay in the past 12 months** | 0.09 | 1.00 | 0.35 | 1.02 | 0.11 | 1.00** | | | 1.26 | | 9.09 | 7.61 | 19.92 | 2.62 | 12.45 |

*p<0.001, **p<0.01

**Table S3** Information on employment and service use at one-year post-baseline period

| Characteristics | Early stage (n=372) | | | | | | | Later stage (n=177) | | | | | | |
| --- | --- | --- | --- | --- | --- | --- | --- | --- | --- | --- | --- | --- | --- | --- |
|  | Non-LICM | | LICM | | Total | | | Non-LICM | | | LICM | | Total | |
|  | N | % | N | % | N | | % | N | % | | N | % | N | % |
| **Employment** |  |  |  |  |  | |  |  | |  |  |  |  |  |
| No | 206 | 59.5 | 20 | 76.9 | 226 | | 60.8 | 103 | | 74.1 | 31 | 81.6 | 134 | 75.7 |
| Yes | 140 | 40.5 | 6 | 23.1 | 146 | | 39.3 | 36 | | 25.9 | 7 | 18.4 | 43 | 24.3 |
| **Psychiatric admission** |  |  |  |  |  | |  |  | |  |  |  |  |  |
| No | 341 | 98.6 | 20 | 76.9 | 361 | | 97.0* | 138 | | 99.3 | 23 | 60.5 | 161 | 91.0* |
| Yes | 5 | 1.5 | 6 | 23.1 | 11 | | 3.0 | 1 | | 0.7 | 15 | 39.5 | 16 | 9.0 |
| **Any psychiatric ER visit** |  |  |  |  |  | |  |  | |  |  |  |  |  |
| No | 341 | 98.6 | 20 | 76.9 | 361 | | 97.0* | 137 | | 98.6 | 27 | 71.1 | 164 | 92.7* |
| Yes | 5 | 1.5 | 6 | 23.1 | 11 | | 3.0 | 2 | | 1.4 | 11 | 29.0 | 13 | 7.3 |
|  |  |  |  |  |  | |  |  | |  |  |  |  |  |
|  | Mean | SE | Mean | SE | Mean | | SE | Mean | | SE | Mean | SE | Mean | SE |
| **Total length of stay (days)** | 0.62 | 0.30 | 5.23 | 2.19 | 1.87 | 0.45* | | 0.22 | | 0.22 | 17 | 5.20 | 3.82 | 1.21* |
| **Number of outpatient visits** | 2.91 | 0.10 | 5.31 | 0.36 | 3.19 | 0.11* | | 2.77 | | 0.20 | 5.68 | 0.40 | 3.40 | 0.20* |

*p<0.001
